# Retraction: Quantifying the effects of co-composting organic biomass mixtures with inorganic amendments to obtain value-added bio-products

**DOI:** 10.1371/journal.pone.0312844

**Published:** 2024-10-24

**Authors:** The PLOS ONE Editors

This article [1] was investigated by PLOS due to indicators that suggest it may be linked to a series of submissions with related integrity concerns.

For this article [1], we noted concerns about the article’s authorship and compliance with the PLOS Data Availability policy. Contrary to the Data Availability statement, the individual-level data underlying the published results were not provided with the paper.

The underlying data were provided by the first author upon editorial request. PLOS and an independent member of the *PLOS ONE* Editorial Board reviewed the article and the underlying data provided. During this review, several additional concerns were raised about the study design, the reported results, and the citations and references listed in this article.

These issues were not resolved in follow-up discussions.

In light of the above concerns that question the reliability of the published results, the *PLOS ONE* Editors retract this article.

RSN did not agree with the retraction. YS, JQ, FH, MMW, ANS, and RN either did not respond directly or could not be reached.
